# Coding variant Met72Thr in the *PEDF* gene and risk of neovascular age-related macular degeneration and polypoidal choroidal vasculopathy

**Published:** 2009-06-02

**Authors:** Hiroaki Bessho, Naoshi Kondo, Shigeru Honda, Shin-ichi Kuno, Akira Negi

**Affiliations:** 1 Department of Surgery, Division of Ophthalmology, Kobe University Graduate School of Medicine, Kobe, Japan; 2 Translational Research Informatics Center, Foundation for Biomedical Research and Innovation, Kobe, Japan; 3 Clinical Genome Informatics Center, Kobe University Graduate School of Medicine, Kobe, Japan

## Abstract

**Purpose:**

Using a candidate-gene approach, a recent case-control study identified a previously unknown association between neovascular age-related macular degeneration (AMD) and the coding Met72Thr variant in the pigment epithelium-derived factor (PEDF) gene in a Taiwan Chinese population. However, a subsequent replication study failed to see this association in a white European population. We noted an important difference in the sample ascertainment scheme between these two studies. The original study did not consider findings of indocyanine green (ICG) angiography for disease classification, which is the only way to obtain a clear image of polypoidal choroidal vasculopathy (PCV) lesions. This suggests that their cohort might include a considerable amount of PCV, given its high prevalence in the Chinese population. In contrast, the replication study intentionally excluded PCV from the case cohort on the basis of ICG angiograms. Therefore, the inconsistent finding might be caused by potential sample heterogeneity between these two studies. In this respect, this association needed to be examined in a case series of clearly defined individuals with neovascular AMD and PCV. The aim of this study was to validate the previously reported association of the *PEDF* Met72Thr variant in a well characterized Japanese population with neovascular AMD and PCV.

**Methods:**

We genotyped the Met72Thr variant (rs1136287) in 116 patients with neovascular AMD, 140 patients with PCV, and 189 control participants in a Japanese population. Genotyping was performed using TaqMan technology. We tested for an association of this variant with neovascular AMD and PCV separately. We also evaluated population stratification in our study cohort.

**Results:**

We found no statistically significant evidence for association between rs1136287 and either neovascular AMD or PCV under any genetic models (trend, genotypic, dominant, and recessive genetic models; p>0.05). Population structure analyses excluded stratification artifact in our study population.

**Conclusions:**

We report a lack of association between the *PEDF* Met72Thr variant and either neovascular AMD or PCV in a Japanese population. We conclude that the Met72Thr variant does not play a significant role in the risk of developing neovascular AMD or PCV.

## Introduction

Age-related macular degeneration (AMD), a leading cause of blindness among older individuals in developed countries [1], is a heterogeneous group of disorders with variable clinical findings [2]. It manifests at an early stage with large drusen and pigmentary abnormalities in the retinal pigment epithelium at the macula. With progression to an advanced stage, it presents with geographic atrophy (dry AMD) or exudative maculopathy (wet/neovascular AMD) as the sequela of choroidal neovascularization (CNV).

Inner choroidal vascular networks ending in polypoidal lesions are the defining feature of polypoidal choroidal vasculopathy (PCV) [3-5], which is now clinically classified into a specific type of AMD [6]. Some clinical and pathological features such as demography [7], pathology [8-10], and manifestation [7] are common between neovascular AMD and PCV; however, important differences are also noted in clinical behavior [3], histopathology [11,12], and response to therapy [13,14]. These commonalities and differences have been a source of much debate as to whether PCV has a common etiology with neovascular AMD or a distinct phenotype reflecting a different etiology [8-12,15]. PCV has a particularly high incidence in Asian populations, accounting for 54.7% of patients with findings suggestive of neovascular AMD in the Japanese population [7] and 24.5% in the Chinese population [16], in contrast to only 8 to 13% in European populations [3]. The phenotypic spectrum of AMD is quite heterogeneous among different ethnicities. Dry AMD is more frequent in European populations than in Asians; the reverse is true for neovascular AMD [17-19].

To date, many studies have reported various gene variants associated with AMD [20,21]. However, findings from most studies have been largely inconclusive because of a lack of consistent replication [20,21]. Thus far, variants in two genomic regions have been consistently reproducible across multiple ethnic groups, including the complement factor H (*CFH*) gene at chromosome 1q32 [22-26] and the *ARMS2* (*LOC387715*)/*HTRA1* locus at 10q26 [27-30]. These two loci are associated with all phenotypes of AMD, including early AMD, dry AMD, and neovascular AMD [25,27]. Genetic susceptibility to PCV is also strongly associated with the *CFH* gene and the *ARMS2*/*HTRA1* locus [31,32], thus indicating that these two loci play a general role in the etiology of variable phenotypes of AMD and PCV. Besides these shared genetic associations, two phenotype-specific associations have been found. These associations are common variants in the elastin gene for PCV [33] and an allelic variant (Leu412Phe) in the toll-like receptor 3 (*TLR3*) gene for dry AMD [34]. From the genetic perspective, both general and phenotype-specific pathways may be implicated in the pathophysiology of AMD and PCV. Therefore, to avoid variable findings across studies, attention to disease classification is a key aspect of genetic studies on AMD, as suggested previously [35].

Lin et al. [36] recently reported a previously unknown association between neovascular AMD and the coding Met72Thr (rs1136287) variant in the pigment epithelium-derived factor (*PEDF*) gene in a Taiwan Chinese population. The authors reported that the minor allele T of rs1136287 was significantly associated with increased disease risk, with an odds ratio (OR) of 3.9 under a recessive disease model [36]. At present, there is no evidence for functional relevance of the Met72Thr variant, nor has any biologic explanation been proposed for this association. Still, there are multiple lines of evidence for the importance of the protein product *PEDF* in the pathogenesis of AMD [37], and thus, *PEDF* is a reasonable candidate gene for the disease.

Mattes et al. [38] subsequently failed to replicate this association in a white European population with neovascular AMD. We noted an important difference in the sample ascertainment scheme between these two studies [36,38]. Lin et al. [36] applied consensus criteria for AMD classification derived from analysis of a white population and did not consider findings of indocyanine green (ICG) angiography for disease classification. ICG angiography is the only way to obtain a clear image of PCV lesions [3]. The work of Lin et al. suggests that their cohort might include a considerable amount of PCV, given its high prevalence in the Chinese population [16]. However, Mattes et al. [38] performed ICG angiography and excluded individuals with PCV from case participants. Since genetic susceptibility to distinct AMD phenotypes seems to be influenced by different genetic factors [33-35], genetic association studies on AMD can be confounded by underlying sample heterogeneity. An example of this situation can be seen in previous studies on *TLR3* for its association with AMD. Edwards et al. [39] examined polymorphisms across *TLR* genes, including the *TLR3* Leu412Phe variant, in a mixed sample of early AMD, dry AMD, and neovascular AMD. This analysis was not restricted to dry AMD, and the authors failed to detect a phenotype-specific association of *TLR3* Leu412Phe with dry AMD. In contrast, Yang et al. [34] selectively studied subgroups of subjects with early AMD, dry AMD, and neovascular AMD and eventually found that the Leu412Phe variant specifically contributes to the risk of developing dry AMD. Similarly, the inconsistent finding of the *PEDF* Met72Thr variant could be due to potential sample heterogeneity between the studies by Lin et al. and Mattes et al. Therefore, this association needed to be examined in a case series of clearly defined individuals with neovascular AMD and PCV. The aim of this study was to validate the previously reported association of the *PEDF* Met72Thr variant in a well characterized Japanese population with neovascular AMD and PCV.

## Methods

### Study participants

This study was approved by the Institutional Review Board at Kobe University Graduate School of Medicine and was conducted in accordance with the Declaration of Helsinki. Written informed consent was obtained from all participants. All case and control participants enrolled in this study (more information about participants in Table 1) were Japanese individuals recruited from the Department of Ophthalmology at Kobe University Hospital in Kobe, Japan. This is largely the same sample set used in our previous studies in which phenotyping criteria were fully described [31-33,40]. In brief, all our neovascular AMD and PCV subjects underwent comprehensive ophthalmic examinations, including ICG angiography, and were defined as individuals with angiographically well delineated lesions of CNV or PCV. Patients with secondary choroidal neovascular diseases such as degenerative myopia, idiopathic CNV, ocular trauma, angioid streaks, and presumed ocular histoplasmosis were excluded from the recruitment. The control participants, who were not related to the case participants, were defined as individuals without macular degeneration and changes such as drusen or pigment abnormalities, and thus, were categorized as having the clinical age-related maculopathy staging system stage 1 [41].

**Table 1:** Characteristics of the study population

	**Neovascular AMD**	**PCV**	**Controls**
Number of participants	116	140	189
Males (%)	78	77	60
Mean age±SD (years)	75±7.2	73±6.9	72±5.8
Age range (years)	57–91	57–86	56–95

A total of 116 patients with neovascular AMD, 140 patients with PCV, and 189 control participants were enrolled in the present study. The gender breakdown, mean age, and age range of each population are shown. Abbreviations: age-related macular degeneration (AMD); polypoidal choroidal vasculopathy (PCV); standard deviation (SD).

### Genotyping

Genomic DNA was extracted from peripheral blood immediately after it was drawn. We genotyped the Met72Thr variant (rs1136287) using TaqMan^®^ SNP Genotyping Assays (Assay ID: C___1841779_20; Applied Biosystems, Foster City, CA) on a StepOnePlus™ Real-Time PCR system (Applied Biosystems), in accordance with the manufacturer’s instructions. For verification, a random 10% of samples were genotyped twice, yielding 100% concordance.

### Statistical analysis

Testing for association was performed using software packages SNPGWA v3.02. Deviations from Hardy–Weinberg equilibrium (HWE) were evaluated using the exact test [42] implemented in SNPGWA. We performed association analyses using the Cochran-Armitage trend test [43] and genotypic (2 degrees of freedom, χ^2^ test), dominant (1 degree of freedom, χ^2^ test), and recessive (1 degree of freedom, χ^2^ test) genetic models. The max(T) permutation procedure implemented in PLINK v1.00 was used with 10,000 iterations to obtain empirical p values [44]. To adjust for age and gender differences between case and control subjects, we conducted logistic regression analyses using JMP version 6.0.3 software (SAS Institute, Cary, NC), assuming a multiplicative codominant genetic model by fitting the number of minor allele carried as an ordinal covariate and age and gender as continuous and categorical covariates, respectively. Power calculations were performed using QUANTO version 1.2 [45]. We had full power to detect the OR reported in the original studies for rs1136287 (OR=3.90 under a recessive genetic model) at p<0.05 for both neovascular AMD and PCV. Assuming an additive genetic model, we had 80% power to detect an association of this variant with ORs ≥1.61 (or ≤0.62) for the neovascular AMD sample and ≥1.56 (or ≤0.63) for the PCV sample.

Linkage disequilibrium (LD) structures across the *PEDF* gene were compared between Chinese and Japanese populations, using genotype data retrieved from the HapMap [46] CHB (Han Chinese in Beijing, China) and JPT (Japanese in Tokyo, Japan) data sets. These retrieved data were loaded into Haploview [47] to estimate LD parameters and identify haplotype blocks.

Hidden population stratification in genetic association studies can generate a spurious positive or negative association [48]. To prevent potential stratification in our study cohort, we evaluated population stratification using STRUCTURE software [49], as described in previous studies [40,50,51]. The following 38 single nucleotide polymorphisms (SNPs), which were randomly distributed along the genome and are not in LD with each other (r^2^<0.04), were used for stratification analysis: rs3818729 (1p13.2), rs696619 (1p21.3), rs9434 (1p36.12), rs1554286 (1q32.1), rs13388696 (2p23.1), rs1042034 (2p24.1), rs10932613 (2q35), rs7641926 (3p26.2), rs2305619 (3q25.32), rs4074 (4q13.3), rs6876885 (5p15.1), rs6459193 (6p11.2), rs3779109 (7p22.1), rs2227667 (7q22.1), rs6468284 (8p12), rs10757278 (9p21.3), rs955220 (9p24.3), rs1927911 (9q33.1), rs4838590 (10q11.22), rs12806 (10q24.2), rs2019938 (11p15.5), rs609017 (11q24.3), rs3912640 (12p13.2), rs2283299 (12p13.33), rs715948 (12q13.3), rs7328193 (13q12.11), rs1048990 (14q13.2), rs911669 (14q32.13), rs16948719 (15q22.31), rs11076720 (16q24.3), rs1051009 (17p13.2), rs1292033 (17q23.1), rs7239116 (18q11.2), rs892115 (19p13.2), rs3826945 (19p13.3), rs844906 (20p11.21), rs2825761 (21q21.1), and rs3884935 (22q13.1). The log likelihood of each analysis at a varying number *K* (the number of populations) was computed from three independent runs (20,000 burn-in and 30,000 iterations). The best estimate of *K* was defined by calculating posterior probabilities *Pr* (*K*=1, 2, 3, 4, or 5) based on the log likelihood, as described by Pritchard et al. [52].

## Results

A total of 116 patients with neovascular AMD, 140 patients with PCV, and 189 control participants participated in the study. Table 1 shows the demographic details of the study population. The *PEDF* Met72Thr (rs1136287) variant showed no significant deviation from Hardy–Weinberg equilibrium in the control participants (p=0.66).

Details of allele and genotype counts and summary statistics for rs1136287 are shown in Table 2. We found no statistically significant evidence for association between rs1136287 and either neovascular AMD or PCV under any genetic models (Table 2). Adjusting for age and gender by logistic regression analyses did not affect the conclusion (age- and sex-adjusted p=0.59 for neovascular AMD; age- and sex-adjusted p=0.33 for PCV, under a multiplicative codominant model).

**Table 2:** Allele and genotype distributions of rs1136287 and the results of association tests

**Status**	**Genotype**			**Allele**	OR trend* **(95% CI)**	**p values (empirical p values)**			
	**CC**	**CT**	**TT**	**T**		**Trend**	**Genotype**	**Dominant**	**Recessive**
Control	56 (0.30)	91 (0.48)	42 (0.22)	175 (0.46)	-	-	-	-
Neovascular AMD	41 (0.35)	52 (0.45)	23 (0.20)	98 (0.42)	0.86 (0.62–1.18)	0.34 (0.35)	0.58 (0.58)	0.30 (0.32)	0.62 (0.67)
PCV	32 (0.23)	78 (0.56)	30 (0.21)	138 (0.49)	1.13 (0.83–1.55)	0.44 (0.47)	0.32 (0.31)	0.17 (0.20)	0.86 (0.89)

The trend p values were obtained by the Cochran-Armitage trend test. Genotypic p values were generated by chi-square tests on 2×3 contingency tables. Tests for the dominant and recessive models were set up as 2×2 contingency table chi-square tests. The dominant model compared a combination of C/T+T/T genotypes to the homozygous C/C. The recessive model compared a combination of C/C+C/T to the homozygous T/T. Empirical p values were generated by 10,000 permutation tests, using the max(T) permutation procedure implemented in PLINK [44]. Asterisk (*) indicates OR trend test. Abbreviations: odds ratio (OR); confidence interval (CI); age-related macular degeneration (AMD); polypoidal choroidal vasculopathy (PCV).

**Figure 1:**
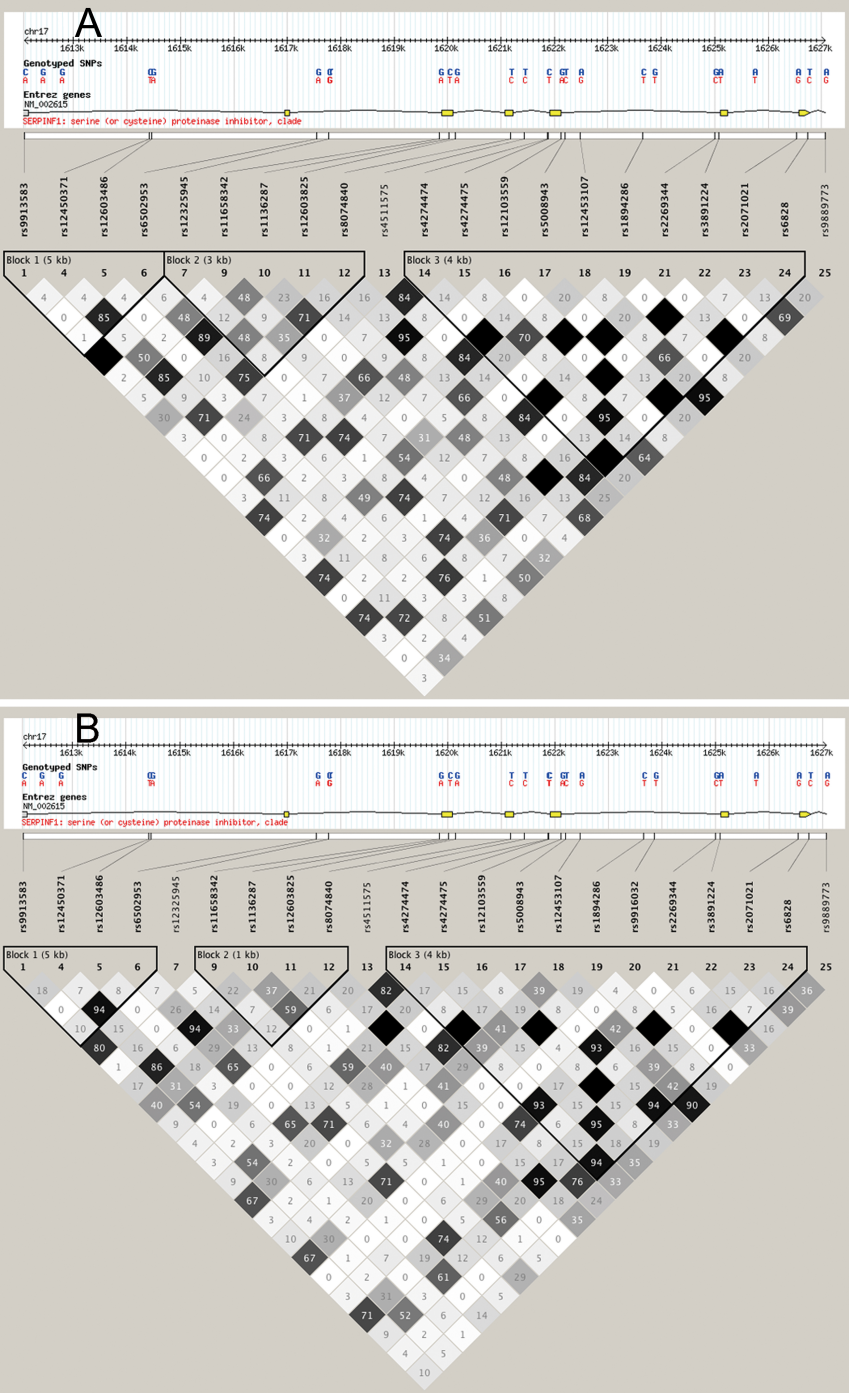
Linkage disequilibrium structure across the pigment epithelium-derived factor gene in Chinese and Japanese populations. Genotype data were retrieved from HapMap CHB (Han Chinese in Beijing, China; **A**) and JPT (Japanese in Tokyo, Japan; **B**) data sets, and linkage disequilibrium (LD) patterns were assessed using Haploview software [47]. Haplotype blocks were determined using the “four-gamete rule” option in this software. Each box provides estimated statistics of the coefficient of determination (r^2^), with darker shades representing stronger LD.

We also explored the possibility that failure to replicate the previously reported association could arise due to differences in LD structure between Chinese and Japanese populations—i.e., if the Met72Thr variant is only a proxy for some other true causative variant, the inconsistency may be due to a difference in LD for a pair of the Met72Thr and causal variants between Chinese and Japanese populations. To this end, we compared LD structures across the *PEDF* gene between Chinese and Japanese populations, using genotype data retrieved from the HapMap [46] CHB and JPT data set. This analysis revealed that the HapMap Chinese (CHB) and Japanese (JPT) populations are highly similar with regard to LD pattern and structure of LD blocks across the *PEDF* gene (Figure 1).

Next, we examined SNPs within *PEDF* in an available data set, the NEI/NCBI dbGAP database. This database provides results of genome-wide association analysis for 395 individuals with AMD and 198 controls from the National Eye Institute Age-Related Eye Disease Study (AREDS). This genome-wide association study investigated seven *PEDF* SNPs, including the SNP rs1136287 (Met72Thr) tested here and six other SNPs (rs8074840, rs4274474, rs2269344, rs3891224, rs2071021, and rs6828). None of these seven SNPs showed significant association with AMD in this analysis (p>0.05).

Hidden population stratification between case and control subjects can be a confounding factor yielding false-positive or false-negative findings [48]. Population stratification was examined by STRUCTURE [49], using 38 unlinked genome-wide SNPs. We found no evidence of stratification in our study cohort, using the formula;

Pr (K=1 > 0.99),

thus indicating that our results did not arise from population stratification.

## Discussion

To validate the recently reported association between the *PEDF* Met72Thr variant and wet AMD in a Taiwan Chinese population [36], we analyzed this variant in Japanese subjects with neovascular AMD and PCV separately, given the possibility that a case population of the original report might be a mixed sample of neovascular AMD and PCV. Despite sufficient power to replicate the originally reported association, we found no statistically significant evidence for association between this variant and either neovascular AMD or PCV.

PEDF is mainly a secreted 50 kDa glycoprotein that is found in various ocular tissues including retinal pigment epithelium and choroid [53,54]. PEDF potently inhibits angiogenesis and plays a pivotal role in maintaining vascular quiescence in the eye [54,55]. Specifically, PEDF has been repeatedly implicated in the pathology of neovascular AMD: CNV is associated with decreased levels of PEDF in an eye with neovascular AMD [56,57], *PEDF* gene transfer in the eye inhibits laser-induced CNV growth and promotes regression of established CNV in a murine model [58,59], and adenoviral vector-mediated intravitreal gene transfer of PEDF seems to help prevent the growth of CNV in patients with neovascular AMD [60]. Thus, from a functional perspective, *PEDF* appeared to be a reasonable candidate for genetic susceptibility to neovascular AMD.

Disease heterogeneity can result in false-positive reporting or can obscure the existence of true associations. Population stratification can also generate inconsistent findings across studies, thus yielding false-positive or false-negative association. To address these concerns, we performed association analysis for both neovascular AMD and PCV because of the unclear definition of the disease phenotype included in the original study [36]. We also examined population stratification in our study cohort and confirmed the lack of stratification. Many other reasons for irreproducibility of initial positive findings have been cited, with failure to exclude chance being considered the most likely explanation for difficulty in replication [61,62]. To protect against a range of confounding factors and to provide greater support for a claim of association, initial positive studies should preferably include multiple replication samples [61,62]. However, Lin et al. did not validate their finding in a second sample, nor did they evaluate potential stratification in their study cohort [36]. Finally, our inability to replicate the previously reported association may result from differences in the structure of LD across the *PEDF* gene region between Chinese and Japanese populations. However, examination of HapMap data [46] revealed that the HapMap Chinese and Japanese populations are similar in LD pattern and structure of LD blocks across the *PEDF* gene (Figure 1). Mattes et al. also [38] failed to replicate the original finding of Lin et al. [36]. Additional support of our negative findings comes from the genome-wide association study on the AREDS cohort. Our experience highlights the importance of replication efforts in genetic association studies of complex human diseases.

In conclusion, we report a lack of association between the *PEDF* Met72Thr variant and either neovascular AMD or PCV in a Japanese population. We conclude that the Met72Thr variant does not play a significant role in the risk of developing neovascular AMD or PCV.

## References

[1] Friedman DS, O'Colmain BJ, Munoz B, Tomany SC, McCarty C, de Jong PT, Nemesure B, Mitchell P, Kempen J, Eye Diseases Prevalence Research Group. (2004). Prevalence of age-related macular degeneration in the United States.. Arch Ophthalmol.

[2] Jager RD, Mieler WF, Miller JW (2008). Age-related macular degeneration.. N Engl J Med.

[3] Ciardella AP, Donsoff IM, Huang SJ, Costa DL, Yannuzzi LA (2004). Polypoidal choroidal vasculopathy.. Surv Ophthalmol.

[4] Yannuzzi LA, Sorenson J, Spaide RF, Lipson B (1990). Idiopathic polypoidal choroidal vasculopathy (IPCV).. Retina.

[5] Yannuzzi LA, Wong DW, Sforzolini BS, Goldbaum M, Tang KC, Spaide RF, Freund KB, Slakter JS, Guyer DR, Sorenson JA, Fisher Y, Maberley D, Orlock DA (1999). Polypoidal choroidal vasculopathy and neovascularized age-related macular degeneration.. Arch Ophthalmol.

[6] Takahashi K, Ishibashi T, Ogur Y, Yuzawa M (2008). Working Group for Establishing Diagnostic Criteria for Age-Related Macular Degeneration. Classification and diagnostic criteria of age-related macular degeneration.. Nippon Ganka Gakkai Zasshi.

[7] Maruko I, Iida T, Saito M, Nagayama D, Saito K (2007). Clinical characteristics of exudative age-related macular degeneration in Japanese patients.. Am J Ophthalmol.

[8] Kikuchi M, Nakamura M, Ishikawa K, Suzuki T, Nishihara H, Yamakoshi T, Nishio K, Taki K, Niwa T, Hamajima N, Terasaki H (2007). Elevated C-reactive protein levels in patients with polypoidal choroidal vasculopathy and patients with neovascular age-related macular degeneration.. Ophthalmology.

[9] Terasaki H, Miyake Y, Suzuki T, Nakamura M, Nagasaka T (2002). Polypoidal choroidal vasculopathy treated with macular translocation: clinical pathological correlation.. Br J Ophthalmol.

[10] Tong JP, Chan WM, Liu DT, Lai TY, Choy KW, Pang CP, Lam DS (2006). Aqueous humor levels of vascular endothelial growth factor and pigment epithelium-derived factor in polypoidal choroidal vasculopathy and choroidal neovascularization.. Am J Ophthalmol.

[11] Nakajima M, Yuzawa M, Shimada H, Mori R (2004). Correlation between indocyanine green angiographic findings and histopathology of polypoidal choroidal vasculopathy.. Jpn J Ophthalmol.

[12] Nakashizuka H, Mitsumata M, Okisaka S, Shimada H, Kawamura A, Mori R, Yuzawa M (2008). Clinicopathologic findings in polypoidal choroidal vasculopathy.. Invest Ophthalmol Vis Sci.

[13] Gomi F, Sawa M, Sakaguchi H, Tsujikawa M, Oshima Y, Kamei M, Tano Y (2008). Efficacy of intravitreal bevacizumab for polypoidal choroidal vasculopathy.. Br J Ophthalmol.

[14] Silva RM, Figueira J, Cachulo ML, Duarte L, Faria de Abreu JR, Cunha-Vaz JG (2005). Polypoidal choroidal vasculopathy and photodynamic therapy with verteporfin.. Graefes Arch Clin Exp Ophthalmol.

[15] Yuzawa M, Mori R, Kawamura A (2005). The origins of polypoidal choroidal vasculopathy.. Br J Ophthalmol.

[16] Liu Y, Wen F, Huang S, Luo G, Yan H, Sun Z, Wu D (2007). Subtype lesions of neovascular age-related macular degeneration in Chinese patients.. Graefes Arch Clin Exp Ophthalmol.

[17] Klein R, Klein BE, Linton KL (1992). Prevalence of age-related maculopathy. The Beaver Dam Eye Study.. Ophthalmology.

[18] Mitchell P, Smith W, Attebo K, Wang JJ (1995). Prevalence of age-related maculopathy in Australia. The Blue Mountains Eye Study.. Ophthalmology.

[19] Oshima Y, Ishibashi T, Murata T, Tahara Y, Kiyohara Y, Kubota T (2001). Prevalence of age related maculopathy in a representative Japanese population: the Hisayama study.. Br J Ophthalmol.

[20] Haddad S, Chen CA, Santangelo SL, Seddon JM (2006). The genetics of age-related macular degeneration: a review of progress to date.. Surv Ophthalmol.

[21] Scholl HP, Fleckenstein M, Charbel Issa P, Keilhauer C, Holz FG, Weber BH (2007). An update on the genetics of age-related macular degeneration.. Mol Vis.

[22] Edwards AO, Ritter R, Abel KJ, Manning A, Panhuysen C, Farrer LA (2005). Complement factor H polymorphism and age-related macular degeneration.. Science.

[23] Haines JL, Hauser MA, Schmidt S, Scott WK, Olson LM, Gallins P, Spencer KL, Kwan SY, Noureddine M, Gilbert JR, Schnetz-Boutaud N, Agarwal A, Postel EA, Pericak-Vance MA (2005). Complement factor H variant increases the risk of age-related macular degeneration.. Science.

[24] Klein RJ, Zeiss C, Chew EY, Tsai JY, Sackler RS, Haynes C, Henning AK, SanGiovanni JP, Mane SM, Mayne ST, Bracken MB, Ferris FL, Ott J, Barnstable C, Hoh J (2005). Complement factor H polymorphism in age-related macular degeneration.. Science.

[25] Magnusson KP, Duan S, Sigurdsson H, Petursson H, Yang Z, Zhao Y, Bernstein PS, Ge J, Jonasson F, Stefansson E, Helgadottir G, Zabriskie NA, Jonsson T, Björnsson A, Thorlacius T, Jonsson PV, Thorleifsson G, Kong A, Stefansson H, Zhang K, Stefansson K, Gulcher JR (2006). CFH Y402H confers similar risk of soft drusen and both forms of advanced AMD.. PLoS Med.

[26] Maller J, George S, Purcell S, Fagerness J, Altshuler D, Daly MJ, Seddon JM (2006). Common variation in three genes, including a noncoding variant in CFH, strongly influences risk of age-related macular degeneration.. Nat Genet.

[27] Cameron DJ, Yang Z, Gibbs D, Chen H, Kaminoh Y, Jorgensen A, Zeng J, Luo L, Brinton E, Brinton G, Brand JM, Bernstein PS, Zabriskie NA, Tang S, Constantine R, Tong Z, Zhang K (2007). HTRA1 variant confers similar risks to geographic atrophy and neovascular age-related macular degeneration.. Cell Cycle.

[28] Fritsche LG, Loenhardt T, Janssen A, Fisher SA, Rivera A, Keilhauer CN, Weber BH (2008). Age-related macular degeneration is associated with an unstable ARMS2 (LOC387715) mRNA.. Nat Genet.

[29] Dewan A, Liu M, Hartman S, Zhang SS, Liu DT, Zhao C, Tam PO, Chan WM, Lam DS, Snyder M, Barnstable C, Pang CP, Hoh J (2006). HTRA1 promoter polymorphism in wet age-related macular degeneration.. Science.

[30] Yang Z, Camp NJ, Sun H, Tong Z, Gibbs D, Cameron DJ, Chen H, Zhao Y, Pearson E, Li X, Chien J, Dewan A, Harmon J, Bernstein PS, Shridhar V, Zabriskie NA, Hoh J, Howes K, Zhang K (2006). A variant of the HTRA1 gene increases susceptibility to age-related macular degeneration.. Science.

[31] Kondo N, Honda S, Ishibashi K, Tsukahara Y, Negi A (2007). LOC387715/HTRA1 variants in polypoidal choroidal vasculopathy and age-related macular degeneration in a Japanese population.. Am J Ophthalmol.

[32] Kondo N, Honda S, Kuno S, Negi A (2009). Coding variant I62V in the complement factor H gene is strongly associated with polypoidal choroidal vasculopathy.. Ophthalmology.

[33] Kondo N, Honda S, Ishibashi K, Tsukahara Y, Negi A (2008). Elastin gene polymorphisms in neovascular age-related macular degeneration and polypoidal choroidal vasculopathy.. Invest Ophthalmol Vis Sci.

[34] Yang Z, Stratton C, Francis PJ, Kleinman ME, Tan PL, Gibbs D, Tong Z, Chen H, Constantine R, Yang X, Chen Y, Zeng J, Davey L, Ma X, Hau VS, Wang C, Harmon J, Buehler J, Pearson E, Patel S, Kaminoh Y, Watkins S, Luo L, Zabriskie NA, Bernstein PS, Cho W, Schwager A, Hinton DR, Klein ML, Hamon SC, Simmons E, Yu B, Campochiaro B, Sunness JS, Campochiaro P, Jorde L, Parmigiani G, Zack DJ, Katsanis N, Ambati J, Zhang K (2008). Toll-like receptor 3 and geographic atrophy in age-related macular degeneration.. N Engl J Med.

[35] Swaroop A, Branham KE, Chen W, Abecasis G (2007). Genetic susceptibility to age-related macular degeneration: a paradigm for dissecting complex disease traits.. Hum Mol Genet.

[36] Lin JM, Wan L, Tsai YY, Lin HJ, Tsai Y, Lee CC, Tsai CH, Tseng SH, Tsai FJ (2008). Pigment epithelium-derived factor gene Met72Thr polymorphism is associated with increased risk of wet age-related macular degeneration.. Am J Ophthalmol.

[37] Zarbin MA (2004). Current concepts in the pathogenesis of age-related macular degeneration.. Arch Ophthalmol.

[38] Mattes D, Haas A, Renner W, Steinbrugger I, El-Shabrawi Y, Wedrich A, Werner C, Schmut O, Weger M (2009). Analysis of three pigment epithelium-derived factor gene polymorphisms in patients with exudative age-related macular degeneration.. Mol Vis.

[39] Edwards AO, Chen D, Fridley BL, James KM, Wu Y, Abecasis G, Swaroop A, Othman M, Branham K, Iyengar SK, Sivakumaran TA, Klein R, Klein BE, Tosakulwong N (2008). Toll-like receptor polymorphisms and age-related macular degeneration.. Invest Ophthalmol Vis Sci.

[40] Kondo N, Honda S, Kuno S, Negi A (2009). Coding variant I62V in the complement factor H gene is strongly associated with polypoidal choroidal vasculopathy.. Ophthalmology.

[41] Seddon JM, Sharma S, Adelman RA (2006). Evaluation of the clinical age-related maculopathy staging system.. Ophthalmology.

[42] Wigginton JE, Cutler DJ, Abecasis GR (2005). A note on exact tests of Hardy-Weinberg equilibrium.. Am J Hum Genet.

[43] Balding DJ (2006). A tutorial on statistical methods for population association studies.. Nat Rev Genet.

[44] Purcell S, Neale B, Todd-Brown K, Thomas L, Ferreira MA, Bender D, Maller J, Sklar P, de Bakker PI, Daly MJ, Sham PC (2007). PLINK: a tool set for whole-genome association and population-based linkage analyses.. Am J Hum Genet.

[45] Gauderman WJ (2002). Sample size requirements for matched case-control studies of gene-environment interaction.. Stat Med.

[46] The International HapMap Project (2003). Nature.

[47] Barrett JC, Fry B, Maller J, Daly MJ (2005). Haploview: analysis and visualization of LD and haplotype maps.. Bioinformatics.

[48] Marchini J, Cardon LR, Phillips MS, Donnelly P (2004). The effects of human population structure on large genetic association studies.. Nat Genet.

[49] Falush D, Stephens M, Pritchard JK (2003). Inference of population structure using multilocus genotype data: linked loci and correlated allele frequencies.. Genetics.

[50] Kubo M, Hata J, Ninomiya T, Matsuda K, Yonemoto K, Nakano T, Matsushita T, Yamazaki K, Ohnishi Y, Saito S, Kitazono T, Ibayashi S, Sueishi K, Iida M, Nakamura Y, Kiyohara Y (2007). A nonsynonymous SNP in PRKCH (protein kinase C eta) increases the risk of cerebral infarction.. Nat Genet.

[51] Yamada K, Gerber DJ, Iwayama Y, Ohnishi T, Ohba H, Toyota T, Aruga J, Minabe Y, Tonegawa S, Yoshikawa T (2007). Genetic analysis of the calcineurin pathway identifies members of the EGR gene family, specifically EGR3, as potential susceptibility candidates in schizophrenia.. Proc Natl Acad Sci USA.

[52] Pritchard JK, Stephens M, Donnelly P (2000). Inference of population structure using multilocus genotype data.. Genetics.

[53] Karakousis PC, John SK, Behling KC, Surace EM, Smith JE, Hendrickson A, Tang WX, Bennett J, Milam AH (2001). Localization of pigment epithelium derived factor (PEDF) in developing and adult human ocular tissues.. Mol Vis.

[54] Chader GJ (2001). PEDF: Raising both hopes and questions in controlling angiogenesis.. Proc Natl Acad Sci USA.

[55] Dawson DW, Volpert OV, Gillis P, Crawford SE, Xu H, Benedict W, Bouck NP (1999). Pigment epithelium-derived factor: a potent inhibitor of angiogenesis.. Science.

[56] Holekamp NM, Bouck N, Volpert O (2002). Pigment epithelium-derived factor is deficient in the vitreous of patients with choroidal neovascularization due to age-related macular degeneration.. Am J Ophthalmol.

[57] Bhutto IA, Uno K, Merges C, Zhang L, McLeod DS, Lutty GA (2008). Reduction of endogenous angiogenesis inhibitors in Bruch's membrane of the submacular region in eyes with age-related macular degeneration.. Arch Ophthalmol.

[58] Mori K, Gehlbach P, Yamamoto S, Duh E, Zack DJ, Li Q, Berns KI, Raisler BJ, Hauswirth WW, Campochiaro PA (2002). AAV-mediated gene transfer of pigment epithelium-derived factor inhibits choroidal neovascularization.. Invest Ophthalmol Vis Sci.

[59] Mori K, Gehlbach P, Ando A, McVey D, Wei L, Campochiaro PA (2002). Regression of ocular neovascularization in response to increased expression of pigment epithelium-derived factor.. Invest Ophthalmol Vis Sci.

[60] Campochiaro PA, Nguyen QD, Shah SM, Klein ML, Holz E, Frank RN, Saperstein DA, Gupta A, Stout JT, Macko J, DiBartolomeo R, Wei LL (2006). Adenoviral vector-delivered pigment epithelium-derived factor for neovascular age-related macular degeneration: results of a phase I clinical trial.. Hum Gene Ther.

[61] Colhoun HM, McKeigue PM, Davey Smith G (2003). Problems of reporting genetic associations with complex outcomes.. Lancet.

[62] NCI-NHGRI Working Group on Replication in Association Studies, Chanock SJ, Manolio T, Boehnke M, Boerwinkle E, Hunter DJ, Thomas G, Hirschhorn JN, Abecasis G, Altshuler D, Bailey-Wilson JE, Brooks LD, Cardon LR, Daly M, Donnelly P, Fraumeni JF, Freimer NB, Gerhard DS, Gunter C, Guttmacher AE, Guyer MS, Harris EL, Hoh J, Hoover R, Kong CA, Merikangas KR, Morton CC, Palmer LJ, Phimister EG, Rice JP, Roberts J, Rotimi C, Tucker MA, Vogan KJ, Wacholder S, Wijsman EM, Winn DM, Collins FS (2007). Replicating genotype-phenotype associations.. Nature.

